# COVID-19: Advice from the Canadian Association of Gastroenterology for Endoscopy Facilities, as of March 16, 2020

**DOI:** 10.1093/jcag/gwaa012

**Published:** 2020-03-27

**Authors:** Frances Tse, Mark Borgaonkar, Grigorios I Leontiadis

**Affiliations:** 1 Practice Affairs Chair, Canadian Association of Gastroenterology, Ontario, Canada; 2 Division of Gastroenterology, Department of Medicine, McMaster University, Hamilton, Ontario, Canada; 3 Endoscopy Chair, Canadian Association of Gastroenterology, Ontario, Canada; 4 Division of Gastroenterology, Department of Medicine, Memorial University, St. John’s, Newfoundland and Labrador, Canada; 5 VP Clinical Affairs, Canadian Association of Gastroenterology, Ontario, Canada

As the number of cases of novel coronavirus disease (COVID-19) rise in Canada and in the rest of the world, and with the designation of COVID-19 as a pandemic by the World Health Organization on March 11, 2020, the Canadian Association of Gastroenterology has issued the following guidance for endoscopy facilities to reduce and delay transmission of COVID-19.

COVID-19 is a rapidly evolving global challenge. As endoscopists and physicians, we have the responsibility of protecting our patients, ourselves and other endoscopy personnel from this infection. To this end, we wish to emphasize the importance of following current guidance and advice from Public Health Agency of Canada on infection prevention and control for COVID-19. It is imperative that all endoscopy facilities, whether they are hospital endoscopy units, out-of-hospital premises or independent health facilities, develop institutional protocols and policies that are consistent with national, provincial and local municipal recommendations for infection control to reduce the spread of COVID-19 to both patients and personnel.

**Figure 1.**
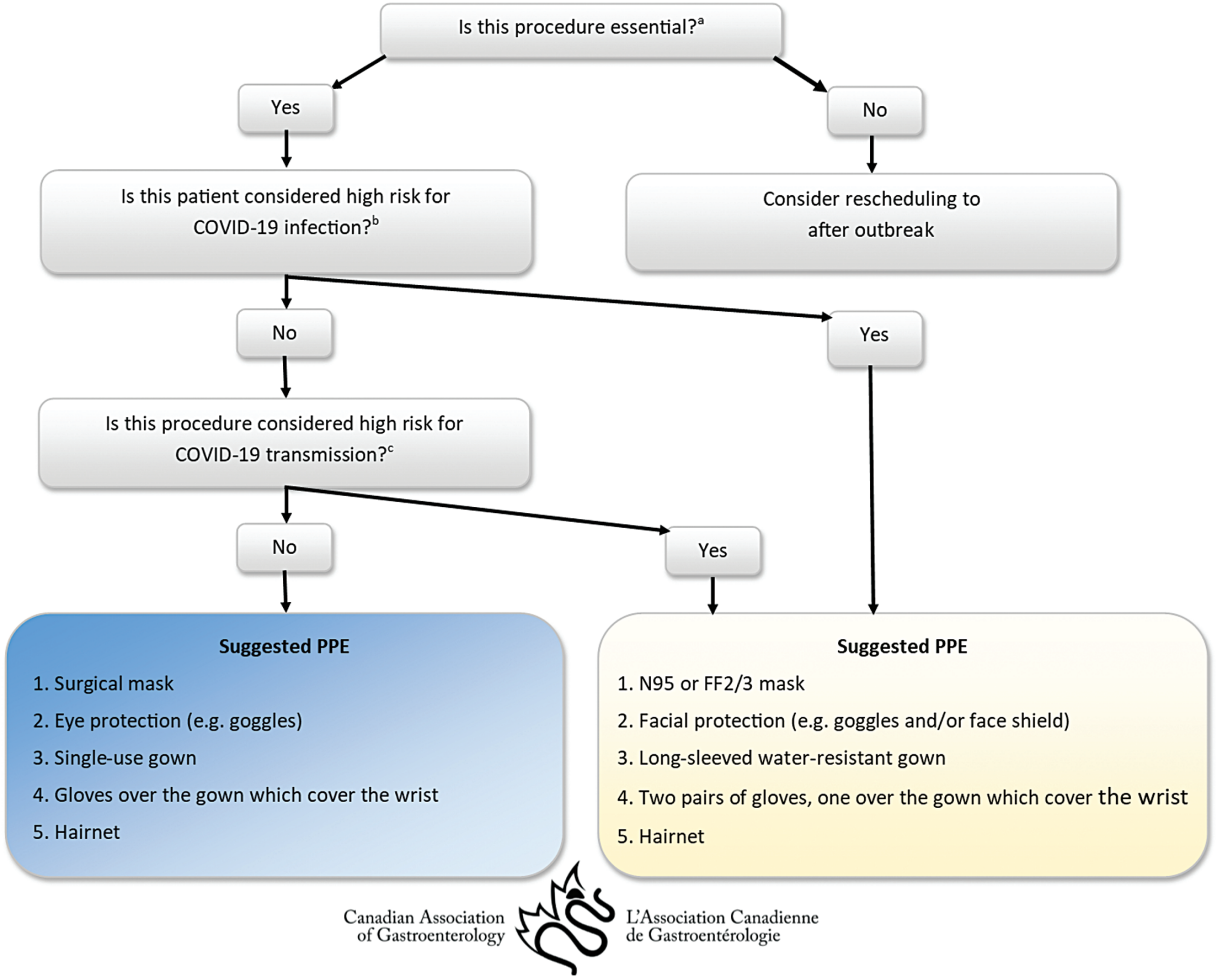
CAG Guidance on infection prevention and control for COVID-19 when performing endoscopic procedures (*as of March 16, 2020)*. Guidance may rapidly change with increasing evidence and understanding of the epidemiology of COVID-19. ^a^Endoscopy facilities should define and repeatedly adjust criteria for essential endoscopic procedures, based on the rate of loss of personnel and resources, and the rapidly evolving local and global epidemiology of COVID-19. Given the limited resources of N95, each institution will have to decide on the criteria for essential GI procedures. If the resources are too low, the institution may have to severely restrict GI procedures to only life-threatening GI bleeding, obstruction of esophagus by food bolus or foreign body, and ascending cholangitis. ^b^Risk assessment and stratification of patients should be repeatedly adjusted based on rapidly evolving local and global epidemiology of COVID-19. ^c^Upper GI procedures are considered high risk procedures for COVID-19 transmission. Insufficient evidence to consider lower GI procedures high-risk procedures for COVID-19 transmission at present.

CAG would like to highlight an important article by Repici et al. who had first hand experience with performing endoscopic procedures during this outbreak.^1^ This article discussed measures, with specific focus on patient management and risk stratification based on procedural types and patient-related risk factors, personal protection equipment (PPE) and process for wearing and removing PPE, which have been implemented in their hospitals to reduce dissemination of COVID-19 infection. It is important to note that the proposed measures are based on current limited understanding of the incubation period, mechanisms of transmission, clinical course and the duration of infectivity. As well, the evidence supporting the use of these measures is largely based on prior experience from other coronaviruses (e.g., SARS-CoV and MERS-CoV). Figure 1 summarizes CAG guidance on infection prevention and control for COVID-19 when performing endoscopic procedures (as of March 16, 2020).

- Risk assessment and stratification of patients should occur prior to any endoscopic procedure. There should be specific triage protocol in place to stratify the risk of COVID-19 upon patient arrival, and ideally prior to arrival as well. This strategy should be repeatedly adjusted based on the rapidly evolving local and global epidemiology of COVID-19.
- All endoscopy facilities should develop standard operating procedures for COVID-19 prevention and control measures in conjunction with local infection control team members, and share these widely among staff members.
- Aerosol-generating medical procedures carry a high risk of COVID-19 transmission. Upper GI procedures are considered high-risk procedures. Therefore, airborne, contact and droplet precautions with appropriate selection and use of PPE including filtering face-piece (e.g., N95, FFP2/3), gloves, gown, facial protection (e.g., goggles and/or face shield) and hairnet are required. Given that community transmission from apparently asymptomatic patients with COVID-19 has already been documented in China and Italy, and now in Canada, and the fact that the prevalence of the disease is likely to be underestimated due to limited testing and restricted criteria for testing in Canada, we suggest regarding all upper GI procedures as high-risk procedures regardless of whether patients are considered low or high risk for COVID-19. This suggestion deviates from Repici et al.^1^
- Although COVID-19 viral RNA has been detected in fecal samples from suspected cases suggesting possible fecal-oral transmission, there is currently insufficient evidence to consider lower GI procedures high-risk procedures for COVID-19 transmission. However, more evidence may change this consideration. The use of appropriate PPE including surgical masks, gloves, gown, eye protection (e.g., goggles) and hairnet are recommended for non-high-risk procedures.
- When any endoscopic procedures are being performed on patients considered to be at high risk for COVID-19 infection, airborne, contact and droplet precautions with appropriate selection and use of PPE including filtering face-piece (e.g., N95, FFP2/3), gloves, gown, facial protection (e.g., goggles and/or face shield) and hairnet. should be used.
- Given potential staff shortages through illness, self-quarantine and isolation, or redeployment, endoscopy facilities should discuss locally and consider whether to reduce nonessential endoscopic activities (e.g., screening and surveillance for polyps, Barrett’s esophagus, etc.) to help reduce or delay the spread of COVID-19 during this outbreak.
- In patients with known or highly suspected COVID-19 infection, endoscopic procedures should only be performed if strongly indicated.
- Given the limited resources of N95, each institution will have to decide on the criteria for ‘essential’ GI procedures. If the resources are too low, the institution may have to severely restrict GI procedures to only life-threatening GI bleeding, obstruction of esophagus by food bolus or foreign body and ascending cholangitis.

The above guidance (Figure 1) reflects the current limited evidence available, but further guidance may be required as the situation rapidly evolves.
